# Supplementary material for: Incorporating male sterility increases hybrid maize yield in low input African farming systems
Source: Commun Biol. 2022 Jul 22;5:729. doi: 10.1038/s42003-022-03680-7 (PMC9307751; doi:10.1038/s42003-022-03680-7)
Supplement: Supplementary file 2 — Supplementary Information [file 42003_2022_3680_MOESM2_ESM.pdf]

Supplementary Figures and Tables

Supplementary Figure 1. Map designating on-farm and on-station yield trial locations.

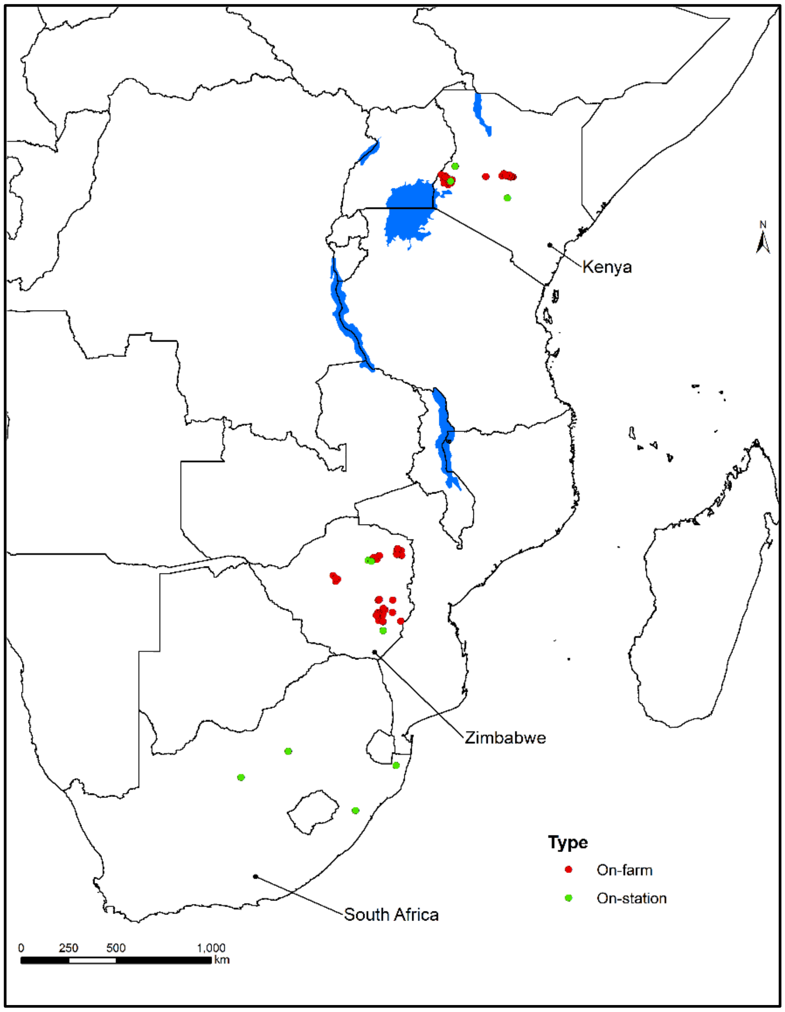

Supplementary Table 1. Number of replicates, hybrid backgrounds and locations in yield trials conducted on-farm (OFT) and on-station (OST) for each country and year.

Country	Year	Type	Locations	Pedigrees	Reps
Kenya	2017	Onfarm	24	4	2
		Onstation	6	4	4
	2018	Onfarm	20	8	2
		Onstation	6	11	4
South Africa	2017-18	Onstation	8	15	4
Zimbabwe	2017-18	Onfarm	17	6	2
		Onstation	7	12	12
	2018-19	Onfarm	18	6	2
		Onstation	6	15	12

Supplementary Table 2. Overview of participants in farmer evaluations.

Year	Region	Mid-season Number of participants			End-season Number of participants			Overall Number of participants		
		Women	Men	Total	Women	Men	Total	Women	Men	Total
2017	Central	86	46	132	131	45	176	217	91	308
	Western	64	73	137	300	261	561	364	334	698
	Total	150	119	269	431	306	737	581	425	1006
	%	56	44	100	58	42	100	58	42	100
2018	Central	275	127	402	295	113	408	570	240	810
	Western	227	193	420	289	172	461	516	365	881
	Total	502	320	822	584	285	869	1086	605	1691
	%	61	39	100	67	33	100	64	36	100
Overall	Total	652	439	1091	1015	591	1606	1667	1030	2697
	%	60	40		63	37		62	38	

---Internal Use---

Supplementary Table 3. Study design for the participatory evaluations.

Criteria No.	Treatment group	Year	Criteria	How important is this criterion? [a]	For each plot, please evaluate the variety for this criterion on a scale of A to E [b]		
					Plot 1	Plot 2	…
1	All	2017 and 2018	Germination/Crop stand				
2	All	2017 and 2018	Height				
3	All	2017 and 2018	Stalk thickness				
4	All	2017 and 2018	Number of cobs per plant				
5	All	2017 and 2018	Cob size				
6	All	2017 and 2018	Barrenness level				
7	All	2017 and 2018	Yield				
8	All	2017 and 2018	Biomass (for fodder)				
9	All	2017 and 2018	Resistance to stalk borer				
10	All	2017 and 2018	Drought resistance				
11	All	2017 and 2018	Foliar disease resistant				
12	All	2017 and 2018	Tillers development				
13	All	2017 and 2018	Early maturing				
14	All	2018 only	Husk cover				
15	All	2018 only	Drooping of the ear				
16	All	2018 only	Cob rot resistance				
17	All	2018 only	Lodging resistance				
14	Treatment 1 and 2 [c]	2017 and 2018	Good tassel formation				
15	Treatment 2 only	2017 and 2018	Amount of pollen shed (good pollination)				
20	All	2017 and 2018	Overall evaluation (note: not an average)				

[a] Codes: 0 = not important, 1 = somewhat important, 2 = important, 3 = very important

[b] Codes: A = like very much, B = like, C = neither like nor dislike, D = dislike, E = dislike very much.

[c] In 2108, four criteria were added.

[c] Note: In the mid-season of 2017, farmers were randomly assigned to three groups: control, treatment 1 and treatment 2; all participants evaluated the varieties on criteria 1 to 13 and 16, both treatment groups also on criterion 14, only those in treatment 2 on criterion 15. In 2018, treatment groups 1 and 2 were merged.

Supplementary Table 4. Expected benefits of fifty-percent non-pollen producing (FNP) in terms of yield, production and economic gain (25 top producing maize countries, including all with 128,000 ha or above). Benefits estimated using a 10% adoption rate of FNP hybrids.

Country	Area harvested (1000 ha)	Yield (kg ha^{-1})	Hybrid adoption rate (% rea)	Yield gain (kg ha-1)	Area in FNP (ha)	Production gain (tonnes)	Economic gain (US$ 1000)	FNP seed needed (tonnes)
Nigeria	4,853	10,155	11.6	192.8	56,299	10,852	1,784	1,407
United Republic of Tanzania	4,101	5,987	40.2	189	164,843	31,149	5,121	4,121
Democratic Republic of Congo	2,680	2,078	9.6	184.9	25,611	4,734	778	640
Angola	2,655	2,271	4.1	185.3	10,884	2,017	332	272
South Africa	2,319	12,510	87.5	212.6	202,899	43,130	7,091	5,072
Ethiopia	2,236	7,360	66	200	147,568	29,506	4,851	3,689
Kenya	2,142	4,014	65	191.4	139,213	26,652	4,382	3,480
Mozambique	1,827	1,654	24.9	185.6	45,484	8,442	1,388	1,137
Malawi	1,685	2,698	65.7	189.8	110,727	21,017	3,455	2,768
Cameroon	1,316	2,345	52.2	190.9	68,737	13,121	2,157	1,718
Zimbabwe	1,191	730	95.4	183.9	113,662	20,900	3,436	2,842
Ghana	1,184	2,306	3.1	191.9	3,672	705	116	92
Benin	1,158	1,510	0	188				
Uganda	1,131	2,964	37.6	195.9	42,524	8,332	1,370	1,063
Mali	1,129	3,625	0	199.5				
Zamboa	1,086	2,395	61.5	193.4	66,789	12,919	2,124	1,670
Burkina Faso	1,019	1,700	3.8	190.2	38,76	737	121	97
Togo	715	887	0.5	187.6	368	69	11	9
Guinea	611	819	6.9	188.2	4,211	793	130	105
Cote d'Ivoire	473	1,006	34.4	193	16,275	3,140	516	407
Chad	342	438	44.6	187.9	15,244	2,864	471	381
Rwanda	296	410	22.4	188.5	6,625	1,249	205	166
Senegal	180	264	10	189	1,791	338	56	45
Madagascar	129	215	0	190.2				
Lesotho	128	100	65.1	184.9	8,313	1,537	253	208
Total top 25		70,441	34.3	191.8	1,255,614	244,204	40,147	31,390
Total sub-Saharan Africa		71,430						

Supplementary Table 5. Economic analysis of FNP hybrids to determine net present value and rate of return; (25 top producing maize countries, including all with 128,000 ha or above). Benefits estimated using a 10% adoption rate of FNP hybrids, compared with estimated investment costs over time.

Year	Year	Cost (1000 USD)	Benefit (1000 USD)	Present value 2020 1000 USD)	Costs discounted (2020 1000 USD)	Benefits discounted (2020 1000 USD)
2010	-11	1000		-2853	2853.1	
2011	-10	1000		-2594	2593.7	
2012	-9	1000		-2358	2357.9	
2013	-8	1000		-2144	2143.6	
2014	-7	1000		-1949	1948.7	
2015	-6	1000		-1772	1771.6	
2016	-5	1000		-1611	1610.5	
2017	-4	1600		-2343	2342.6	
2018	-3	1600		-2130	2129.6	
2019	-2	1600		-1936	1936	
2020	-1	1600		-1750	1760	
2021	0	1250		-1250	1250	
2022	1	1250	4015	2513.4	1136.4	3650
2023	2	1250	8029	5602.8	1033.1	6636
2024	3	1250	12044	8109.8	989.1	9049
2025	4	800	16059	10422	546.4	10968
2026	5	500	20074	12153.7	310.5	12464
2027	6	300	24088	13427.9	169.3	13597
2028	7	100	28103	14370	51.3	14421
2029	8		32118	14983.2		14983
2030	9		36132	15323.7		15324
2031	10		40147	15487.5		15478
2032	11		40147	14071.3		14071
2033	12		40147	12792.1		12792
2034	13		40147	11629.2		11629
2035	14		40147	10572		10572
2036	15		40147	9610.9		9611
2037	16		40147	8737.2		8737
2038	17		40147	7942.9		7943
2039	18		40147	7220.8		7221
2040	19		40147	6564.4		6564
Net present value (NPR)				176.829	28883	205712
Internal rate of return (IRR)				0.26		
Benefits				205712		
Costs				2883		
BC				7.12		
