# Supplementary material for: Incorporating male sterility increases hybrid maize yield in low input African farming systems
Source: Commun Biol. 2022 Jul 22;5:729. doi: 10.1038/s42003-022-03680-7 (PMC9307751; doi:10.1038/s42003-022-03680-7)
Supplement: Supplementary file 3 — Reporting Summary [file 42003_2022_3680_MOESM3_ESM.pdf]

nature portfolio

Corresponding author(s): Michael Olsen

Last updated by author(s): Jun 9, 2022

Reporting Summary

Nature Portfolio wishes to improve the reproducibility of the work that we publish. This form provides structure for consistency and transparency in reporting. For further information on Nature Portfolio policies, see our Editorial Policies and the Editorial Policy Checklist.

Statistics

For all statistical analyses, confirm that the following items are present in the figure legend, table legend, main text, or Methods section.

n/a	Confirmed	
☐	☒	The exact sample size (*n*) for each experimental group/condition, given as a discrete number and unit of measurement
☐	☒	A statement on whether measurements were taken from distinct samples or whether the same sample was measured repeatedly
☐	☒	The statistical test(s) used AND whether they are one- or two-sided<br>*Only common tests should be described solely by name; describe more complex techniques in the Methods section.*
☒	☐	A description of all covariates tested
☒	☐	A description of any assumptions or corrections, such as tests of normality and adjustment for multiple comparisons
☐	☒	A full description of the statistical parameters including central tendency (e.g. means) or other basic estimates (e.g. regression coefficient) AND variation (e.g. standard deviation) or associated estimates of uncertainty (e.g. confidence intervals)
☐	☒	For null hypothesis testing, the test statistic (e.g. *F*, *t*, *r*) with confidence intervals, effect sizes, degrees of freedom and *P* value noted<br>*Give P values as exact values whenever suitable.*
☒	☐	For Bayesian analysis, information on the choice of priors and Markov chain Monte Carlo settings
☒	☐	For hierarchical and complex designs, identification of the appropriate level for tests and full reporting of outcomes
☐	☒	Estimates of effect sizes (e.g. Cohen's *d*, Pearson's *r*), indicating how they were calculated

Our web collection on statistics for biologists contains articles on many of the points above.

Software and code

Policy information about availability of computer code

Data collection	CSPro 7.0.2 in 2017 and CSPro 7.1.2 in 2018 were used for farmer interview data collection. Excel spreadsheets were used for plot datacollection of field trials
Data analysis	STATA 15 was used for analysis of farmer preference data; ASREML-r v3 was used for analysis of crop phenotypic data

For manuscripts utilizing custom algorithms or software that are central to the research but not yet described in published literature, software must be made available to editors and reviewers. We strongly encourage code deposition in a community repository (e.g. GitHub). See the Nature Portfolio guidelines for submitting code & software for further information.

Data

Policy information about availability of data

All manuscripts must include a data availability statement. This statement should provide the following information, where applicable:

- Accession codes, unique identifiers, or web links for publicly available datasets
- A description of any restrictions on data availability
- For clinical datasets or third party data, please ensure that the statement adheres to our policy

The data that support the findings of this study are available at https://data.cimmyt.org/dataset.xhtml?persistentId=hdl:11529/10548515; figures 1 and 2 have associated raw data; no personally identifiable information is included in the data set

March 2021

Field-specific reporting

Please select the one below that is the best fit for your research. If you are not sure, read the appropriate sections before making your selection.

☒ Life sciences ☐ Behavioural & social sciences ☐ Ecological, evolutionary & environmental sciences

For a reference copy of the document with all sections, see nature.com/documents/nr-reporting-summary-flat.pdf

Life sciences study design

All studies must disclose on these points even when the disclosure is negative.

Sample size	No sample size calculation was performed. The primary aim of the study was to determine if the FNP trait provided benefit to farmers at yield levels below 4 t/ha and ideally below 3 t/ha, since national maize yield levels for the majority of Africa South of the Sahara are below 3 t/ha. The experiments were designed to sample a large number of farms in an attempt to ensure there was adequate representation of low-yielding, stressful conditions sampled. Since heritability of grain yield under highly stressful on-farm conditions tends to be lower than under higher yielding, managed conditions, the experiment was focused on obtaining as much on-farm data as possible within the budget constraints of the project.
Data exclusions	Plots with obvious signs of termite or cattle damage, or with high root or stalk lodging scores, were dropped from the analysis. Outliers were categorized as more than 4 standard deviations from the mean. These were identified during analysis and the original data and residual plots reviewed to determine if these values were truly outside of the normal distribution, in which case they were removed.
Replication	In order to ensure reproducibility of results, the experimental design focused on maximizing the number of paired-comparisons of FNP vs PP over hybrid backgrounds, replicates, environments, and years. The study explored the effect of FNP in 26 different hybrid backgrounds and compared FNP with PP hybrids in 112 environments. The estimate of a 200 kg/ha advantage of FNP hybrids represents a highly significant difference which should be able to be replicated in various genetic backgrounds in a range of field conditions likely to be encountered by Africa smallholder farmers.
Randomization	All entries were randomized within replicates and within whole plots. The split-plot randomization restriction was employed to improve control of micro-environmental (spatial) field variation by forcing FNP and PP versions of each hybrid to be tested in adjacent plots. In each case the order of the two treatments (FNP or PP) within hybrid background (whole-plot treatment) was random.
Blinding	Data collection was performed using fieldbooks without treatment information available to data collectors. The phenotype of the FNP trait is visible at flowering time but much less identifiable at harvest time. For within-plot data collected, blinding was not possible since individual plants had to be identified and measured based on their classification as either male-sterile or male-fertile.

Reporting for specific materials, systems and methods

We require information from authors about some types of materials, experimental systems and methods used in many studies. Here, indicate whether each material, system or method listed is relevant to your study. If you are not sure if a list item applies to your research, read the appropriate section before selecting a response.

Materials & experimental systems

n/a	Involved in the study	
☒	☐	Antibodies
☒	☐	Eukaryotic cell lines
☒	☐	Palaeontology and archaeology
☒	☐	Animals and other organisms
☐	☒	Human research participants
☒	☐	Clinical data
☒	☐	Dual use research of concern

Methods

n/a	Involved in the study	
☒	☐	ChIP-seq
☒	☐	Flow cytometry
☒	☐	MRI-based neuroimaging

Human research participants

Policy information about studies involving human research participants

Population characteristics	We had 2,697 participants attending the participatory evaluations. Women were the majority at 62%. The age of the participants ranged from 17 years to 88 years. They on average had 17 years of farming experience and an average of eight years of formal education, indicating primary school completion. Most of them owned land with an average of 0.85 hectare where about half of it was cultivated with maize. They mostly practiced mixed farming where besides crop cultivation, about three-quarters of them owned cattle. Their average cash income in the past year was roughly $1000 of which about half came from their farming activities.
Recruitment	For each site selected for participatory evaluations, neighboring farmers were identified and invited to participate through farmer groups, local administration, and extension officers. This was mainly through general mobilization. It was beyond us to

select who participates (and who does not) from the general community and as such participation was purely self-selection. Since most of the trials were large and involved different treatments, once the participants arrived at the evaluation sites, they were randomly assigned into respective replications and treatments. We used a systematic "every-other-person to arrive" approach to do this. This controls for self-selection into treatments bias.

Ethics oversight

The study protocols were approved by the Social Economics and Global Maize Programs of the International Maize and Wheat Improvement Center (CIMMYT).

Note that full information on the approval of the study protocol must also be provided in the manuscript.
